# Case of Chronic Rheumatism, Treated by the External Application of Veratria

**Published:** 1839-02-16

**Authors:** James F. Latta

**Affiliations:** Chester County, (Pa.,)


*Case of Chronic Rheumatism, treated by the external application of Veratria.* By JAMES F. LATTA, M. D.

J. B., Esq., had been the subject of chronic rheumatism, of a very painful character, for fifteen years. The part affected was the right lower extremity. All the muscular and ligamentous structures contributing to the motion and formation of the knee and hip-joints, appeared to have been involved in the disease. The head of the femoral bone had undergone a partial dislocation, from the contraction of the muscles inserted below it. The flexor-tendons of the leg were also hardened and contracted—while the balance of the entire limb was very much diminished, from atrophy of almost every portion of its structure. The pain attending this state of things was described as unremitting, and, occasionally, very intense. For many years the patient was unable to lie upon the right side, or to sleep with the limb in a flexed position. If, during the night, he unconsciously turned upon the affected side, or if the limb assumed any other than a straight posture, he was soon awakened by a dull, gnawing pain, which was relieved by frictions with the bare hand, or with flannels. A false step, throwing the weight of the body upon the diseased member, or slight exercise upon foot, is represented to have been followed by pain, particularly about the knee-joint, almost beyond endurance.

In January, 1838, I received, through the "Select Medical Library," Dr. Turnbull's remarks on *veratria*, and other medicinal remedies of a cognate character. The flattering results of Dr. T.'s experience with the article in question, induced me to propose a trial of its virtues to the gentleman whose case has been detailed.

My proposal was acceded to with little prospect of relief,—for all the resources of regular practice, the *infallible* remedies of empiricism, and the incantations of magic, had hitherto proved to be unavailing resorts. Agreeably to the directions of Turnbull, I prepared an ointment consisting of fifteen grains of veratria to an ounce of lard—a small portion of which, about the bulk of a chesnut, was directed to be rubbed into the affected part, every night, before going to bed.

Very sensible relief was obtained from the first application. The frictions had not been long continued until that peculiar sensation was perceived, which is described by Dr. Jackson, of Philadelphia, as closely resembling that we experience when a limb is said to be asleep. Unless this result follow the use of the ointment, no advantage, according to Dr. Turnbull, may be expected from its application. The treatment was continued for several successive nights—intermitted and resumed for two or three weeks—when I had the satisfaction of learning that my patient enjoyed, what he had not done for many years, entire exemption from pain.

It is now about ten months since the veratria was last applied. The late sufferer sleeps soundly on either side, and with the limb in either the flexed or straight position. The latter is still shortened, from the muscular contraction alluded to above, and continues of diminished size. *But the pain is gone*—an event to which the subject of these remarks had long ceased to look forward.

*Chester County*, (*Pa.*,) February, 1839.

